# Myelin, white matter, and social deficits in autism spectrum disorder

**DOI:** 10.4103/NRR.NRR-D-25-00366

**Published:** 2025-10-30

**Authors:** Omri Kimchi-Feldhorn, Ariel Nir Sade, Boaz Barak

**Affiliations:** 1 The Sagol School of Neuroscience, Tel Aviv University, Tel Aviv, Israel; 2 The School of Psychological Sciences, Tel Aviv University, Tel Aviv, Israel

**Keywords:** autism spectrum disorder, myelination, neurodevelopment, social behavior, white matter

## Abstract

Autism spectrum disorder is a neurodevelopmental disorder characterized by social interaction challenges, restricted and repetitive behaviors or interests, and communication difficulties. Emerging evidence suggests that disruptions in myelin, the fatty substance that insulates nerve fibers, may play a significant role in shaping the behavioral characteristics observed in individuals with autism spectrum disorder, particularly those related to social behavior. This article provides an overview of current understanding of the interplay between white matter and myelin deficits, social behavior, and autism spectrum disorder. As such, it aims to deepen our understanding of the underlying mechanisms of autism spectrum disorder and potentially contribute to the development of more targeted interventions and support strategies for individuals affected by the disorder.

## Introduction

Autism spectrum disorder (ASD) is a complex neurodevelopmental disorder (NDD) characterized by challenges in social interaction, communication difficulties, repetitive patterns of behavior, and restricted interests (American Psychiatric Association, 2013). Symptoms are usually recognized in the first 2–3 years of life, and ASD is considered a lifelong disorder that continues to affect development and functioning across the lifespan. Clinically, its presentation is highly heterogeneous, with individuals showing a wide range of symptom profiles and severity levels, leading to variable developmental trajectories and outcomes (Halliday et al., 2024). Diagnosis is based on behavioral criteria (DSM-5) and entails confirming the core symptoms across multiple contexts, as there are no definitive biomarkers. Although the precise pathophysiology of ASD remains elusive, several cellular and molecular alterations have been implicated, including abnormalities in neuronal connectivity, synaptic formation and function (Gilbert and Man, 2017), as well as disruptions in glial cells such as oligodendrocytes (Galvez-Contreras et al., 2020), astrocytes (Cano et al., 2024), and microglia (Bar and Barak, 2019; Takanezawa et al., 2021). Notably, these cellular substrates have significant implications for the social deficits frequently observed in ASD, prompting increased research into their specific roles. The precise causes of ASD remain largely elusive, although it is known to have a strong genetic component, with numerous genes and genetic variations implicated in its development and a wide range of phenotypes (Huguet et al., 2013).

The impaired social behavior associated with ASD pathology encompasses a wide range of cognitive and emotional processes that are crucial for forming and maintaining social relationships, interpreting social cues, and engaging in reciprocal interactions (Barak and Feng, 2016). Individuals with ASD often struggle with these fundamental aspects of social behavior, experiencing difficulties in understanding and responding appropriately to social cues, expressing empathy, and establishing meaningful connections with others (Barak and Feng, 2016). Researchers have increasingly focused on understanding the role of myelin deficits in shaping the behavioral characteristics associated with the disorder, particularly those related to social behavior (Ameis and Catani, 2015).

This review delves into the current scientific literature to explore the relationship between myelin alterations, social behavior, and ASD. By elucidating the potential implications of disrupted myelination on social cognitive processes, we seek to contribute to the growing body of knowledge surrounding ASD, ultimately paving the way for improved diagnostic tools, targeted interventions, and enhanced support for individuals affected by this condition.

## Search Strategy

The literature search was conducted using the following bibliographic citation databases: PubMed (via NCBI platform), Scopus (Elsevier), and Google Scholar. Searches were carried out between January 2024 and May 2025. The search terms included combinations of the following keywords, but not only: “autism spectrum disorder,” “ASD,” “white matter,” “myelination,” “social behavior,” “oligodendrocytes,” “animal models,” and “human neuroimaging.” Additional references were identified through manual screening of reference lists from key articles.

## Interplay between Social Behavior and White Matter

The roles of specific brain regions in mediating social behavior have been deeply researched in humans (Harre, 1972; Adolphs, 2003; Ebstein et al., 2010), resulting in the characterization of what is known as the human social brain. This nexus includes both cortical and subcortical regions, among others, the amygdala, orbital frontal cortex, temporal cortex, the medial prefrontal cortex (mPFC), anterior insula, anterior cingulate cortex (ACC), inferior frontal gyrus, and superior temporal sulcus (Adolphs, 2009; Tavares et al., 2015; Barak and Feng, 2016; Chen and Hong, 2018; Gangopadhyay et al., 2021; Mei et al., 2023; Sato et al., 2023). Such long-range cortico-subcortical projections facilitate the integration of emotional, motivational, and contextual information, enabling the fine-tuning of social interactions and adaptive responses.

Recent studies highlight that the human cerebellum plays a key role in social cognition, with these lobules consistently active in neuroimaging tasks involving mentalizing (inferring others’ intentions) and emotional processing (Van Overwalle et al., 2020; Ma et al., 2023). Mechanistic models propose that cerebellar circuits help learn and predict social action sequences, thereby facilitating smooth social interactions and cooperation (Van Overwalle et al., 2020).

The social brain in humans has three main neural networks: the face perception network (Duchaine and Yovel, 2015; Grill-Spector et al., 2017), the mirroring network (Bonini, 2017; Perry et al., 2018), and the mentalizing network (Baetens et al., 2014; Wang et al., 2021). White matter (WM) tracts facilitate communication between the different brain regions that encompass these networks, enabling the integration and processing of social information, such as facial expressions, social cues, and emotions (Becchio et al., 2012; Bombari et al., 2013). It can be deduced that disruption in myelination, and thus in WM integrity in these networks, would lead to improper neural conduction and impair neural circuit synchronicity, thereby resulting in irregular social behavior (Usui, 2024).

While rodent models do not directly replicate the complexity of human-specific social cognitive processes such as face perception or mentalizing, analogous neural circuits involving homologous brain regions have been identified. Specifically, rodent studies often focus on conserved subcortical-limbic circuits, including the amygdala, hypothalamus, prefrontal cortex, hippocampus, and ventral striatum, which mediate fundamental aspects of social behaviors like social recognition, motivation, reward processing, and emotional regulation (Barak and Feng, 2016; Ko, 2017; Kietzman and Gourley, 2023). Although exact network classifications differ between humans and rodents, functional conservation in these key circuits enables meaningful translational insights. Rodent findings about connectivity, excitability, and plasticity within these conserved circuits provide valuable mechanistic clues that help interpret and guide further human neuroimaging studies, ultimately improving our understanding of social behavior deficits in ASD.

Importantly, disruptions in the integrity and synchronization of these circuits, possibly resulting from abnormalities in WM connectivity, are thought to contribute significantly to the social deficits observed in ASD. Indeed, as the main conductive agent of the nervous system, WM is paramount in the synchronization of these brain areas (Pajevic et al., 2014).

WM consists of bundles of axons, or nerve fibers, connecting neurons across various brain regions to form functional circuits. The characteristic white color arises from the presence of myelin (Fields, 2010), a fatty substance produced by myelinating oligodendrocytes (OLs). Myelin forms a protective sheath around nerve fibers and plays a vital role in facilitating rapid and accurate communication by enhancing axonal conduction between neurons within the brain (Fields, 2008; Domingues et al., 2016; Nir and Barak, 2021). Myelin is best known for its role in accelerating action potential conduction, but it also plays a vital part in coordinating neural activity across the brain. By modulating conduction velocity along axonal pathways, myelin ensures the precise timing of spike transmission and supports synchronization between spatially distributed brain regions, which is essential for coherent network function. Myelin also provides long-term metabolic and trophic support to axons through OL-mediated delivery of energy substrates, crucial for axonal maintenance and survival (Nave, 2010; Simons and Nave, 2015; Sade et al., 2026). In addition, evidence highlights activity-dependent myelination as a key mechanism of experience-driven plasticity, allowing adaptive remodeling of WM in response to learning, environmental stimuli, and social interactions (Fields, 2015). Furthermore, disruptions in myelin integrity have been associated not only with conduction failure, but also with maladaptive circuit stabilization, immune dysregulation, and increased vulnerability to neurodegeneration. Together, these multifaceted roles underscore myelin’s importance not only as a passive insulator, but as an active regulator of neural network function, plasticity, and resilience.

During human brain development, myelination primarily occurs after birth and peaks during childhood, continuing into early adulthood (Tomassy et al., 2016). Therefore, the processes of myelination and oligodendrogenesis emerge as critical events that significantly shape various neuroanatomical structures. This critical period is also rife with social stimulus, and as such is inherently linked with the proper development of the emerging social brain (El Waly et al., 2014; Atzil et al., 2018).

Disruptions in WM and myelin development and function have been implicated in various neurological and neuropsychiatric disorders, such as multiple sclerosis (Lassmann, 2018), schizophrenia (Najjar and Pearlman, 2015), Williams syndrome (Barak et al., 2019; Nir and Barak, 2021; Nir Sade et al., 2023), ASD (Ameis and Catani, 2015; Phan et al., 2020; Fischer et al., 2024), and others (Koshiyama et al., 2020).

While multiple sclerosis, for instance, is involved with active demyelination because of an autoimmune response, ASD appears to be characterized by aberrant myelin development. This atypical myelination in individuals with ASD may contribute to variations in fiber thickness across neural networks, potentially persisting from childhood into adulthood (Bercury and Macklin, 2015; Gilbert and Man, 2017).

Conversely, favorable interpersonal interactions in humans and an enriched social environment in animal models have been associated with enhanced WM connectivity, with effects ranging from increased myelinated axon numbers, thicker myelin sheaths, and improvement in motor tasks, suggesting that social engagement can contribute to the development of healthy WM paths (Yang et al., 2012; Campolongo et al., 2018; Goldstein et al., 2021; Gao et al., 2022). Conversely, adverse social experiences or social deprivation can negatively impact WM development in humans and animal models, potentially affecting information transmission and axonal conduction between brain regions connected to social cognition and emotional regulations (Liu et al., 2012, 2020; Makinodan et al., 2012; Toritsuka et al., 2015; Barak and Feng, 2016; Bonnefil et al., 2019; Flinkenflügel et al., 2023). However, recent evidence suggests that social stress in mice can also lead to increased myelination in specific brain regions (Poggi et al., 2022). These findings underscore the complexity of experience-dependent myelin plasticity, which does not uniformly correspond to positive or negative outcomes. For example, excessive or ectopic myelination in stress-related circuits, such as those involving the amygdala or hippocampus, may contribute to the consolidation of maladaptive emotional memories or heightened anxiety-like behaviors. This suggests that increased myelination in certain contexts could reinforce aversive or pathological network states, emphasizing the importance of circuit specificity and timing in interpreting WM changes related to social experience.

**Figure 1:**
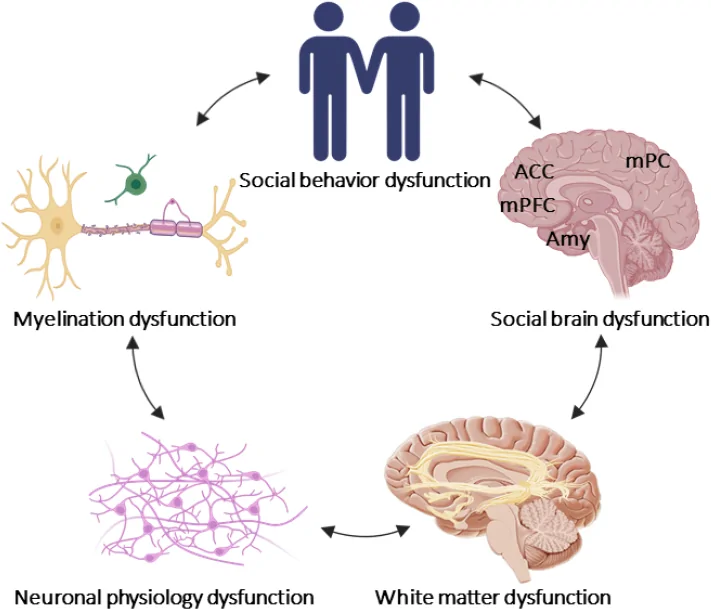
Interplay between social behavior, myelination, and white matter. Disrupted myelin development or abnormalities in myelination properties impede the effective transmission of neural signals through white matter tracts connecting brain regions involved in social cognition and communication. This disruption can impair social behavior, which may, in turn, exacerbate myelin and white matter deficits. Created with BioRender.com. ACC: Anterior cingulate cortex; Amy: amygdala; mPC: medial parietal cortex; mPFC: medial prefrontal cortex.

Several studies in humans have shown weakened social reward in ASD, which could lead to further social aversion and isolation, increasing the detrimental effects on WM and brain connectivity (Cox et al., 2015; Pellissier et al., 2018; Supekar et al., 2018; Baker et al., 2020; Keifer et al., 2021). A question emerges – does the fundamental problem lie in developmental deficits of myelin and WM, leading to the problems with the proper emergence of the social brain structures? Or is it a by-product of other mechanisms underlying the disorder? Either may facilitate a positive feedback loop, leading to further avoidance of social interactions – further weakening synaptic and neuronal connection (Crafa and Nagel, 2020; Shamay-Tsoory, 2022). Both avenues highlight the intrinsic importance of WM in health and NDDs (**Figure 1**).

## White Matter and Myelin Deficits in Autism Spectrum Disorder

The interplay between WM properties and social behavior, as demonstrated in typically developed (TD) individuals in the previous section, can also be demonstrated by studying neuropathology associated with social behavior dysfunction, as in ASD (**Figure 1**).

Postmortem studies indicate that oligodendrogenesis and myelination processes are disrupted in individuals with ASD, but these alterations show region- and context-specific variability, reflecting the heterogeneous nature of the disorder. Proteomic analyses have revealed reduced expression of myelin-related proteins in the prefrontal cortex (Broek et al., 2014) and a decreased fraction of OL-derived RNAs in the frontal and temporal cortex, as well as in the cerebellar vermis of individuals with ASD compared to TD (Phan et al., 2020). In contrast, myelin-related protein expression appears increased in the cerebellum (Broek et al., 2014), as well as myelin-related gene expression in the cerebellum (Zeidán-Chuliá et al., 2016), pointing to potential region-specific compensatory mechanisms or divergent trajectories.

**Figure 2:**
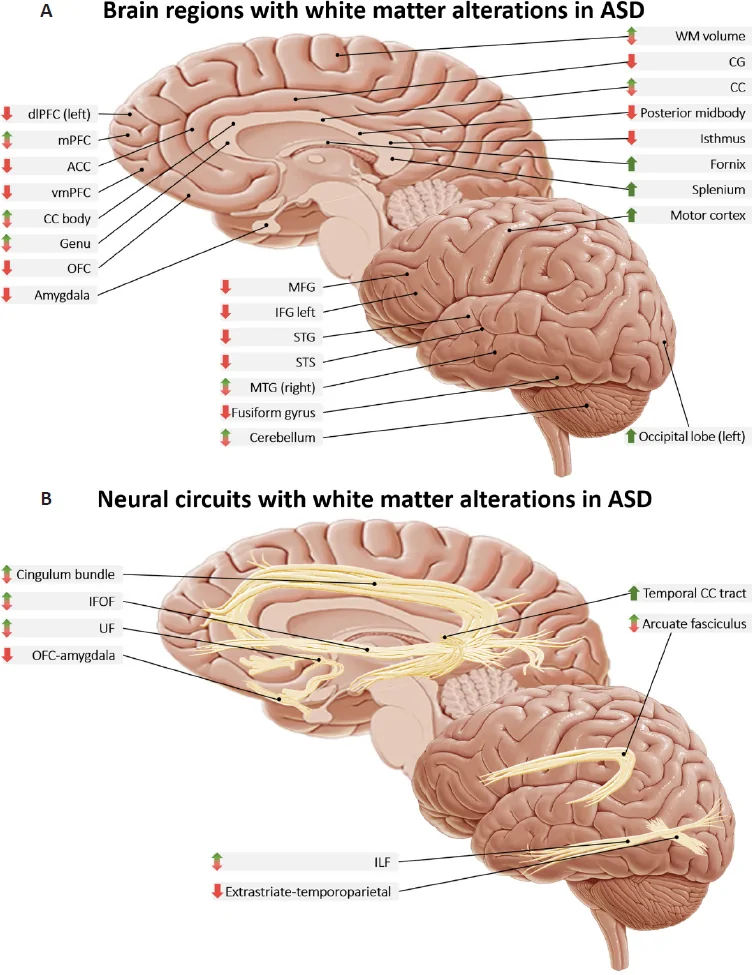
Brain regions and neural circuits presenting white matter (WM) abnormalities in autism spectrum disorder (ASD). WM reductions are marked with red arrows, while elevations of WM are marked with green arrows. Some areas show contradictory findings and are marked with both colors. (A) WM-altered brain regions. In general, WM volume in the autistic brain shows contradictory findings. Frontal region aberrations are noted in the dorso-lateral prefrontal cortex (dlPFC), medial prefrontal cortex (mPFC), anterior cingulate cortex (ACC), ventro-medial prefrontal cortex (vmPFC), and orbitofrontal cortex (OFC). Corpus callosum (CC)-specific regions include the body, genu, posterior midbody, isthmus, and splenium. Limbic areas affected are the amygdala, fornix, and cingulate gyrus (CG). Abnormal gyri include the middle frontal gyrus (MFG), left inferior frontal gyrus (IFG), superior temporal gyrus (STG), right medial temporal gyrus (MTG), and fusiform gyrus. In addition, superior temporal sulcus (STS), cerebellum, and the left occipital lobe present WM abnormalities. (B) WM tracts and pathways presenting changes in ASD include the uncinate fasciculus (UF), inferior fronto-occipital fasciculus (IFOF), inferior longitudinal fasciculus (ILF), arcuate fasciculus, temporal CC tract, and cingulum bundle. In addition, tracts connecting the amygdala and OFC. Created with BioRender.com.

Structural evidence further supports this heterogeneity (**Figure 2**). Adults with ASD exhibit thinner myelin sheaths and increased density of thin axons in the orbitofrontal cortex and subgenual ACC (Zikopoulos and Barbas, 2013). Longitudinally, axonal development in the ACC differs from typical development: while thick, long-range axons decline with age, short, thin axons increase in frequency in ASD individuals compared to TD (Zikopoulos et al., 2018). Consistently, thinner myelinated axons have also been observed in layer 1 of the lateral PFC in ASD brain samples (Trutzer et al., 2019).

Together, these findings suggest that ASD involves both reductions and atypical patterns of myelination that vary across brain regions and developmental stages.

While these studies have shown interesting results in the postmortem brain of varied individuals with ASD, advances in magnetic resonance imaging (MRI) technology have allowed further research into brain structure and function *in vivo*.

Recurring findings from volumetric MRI studies suggest that the brains of individuals with ASD undergo significant growth in early infancy (Shen et al., 2013; Hazlett et al., 2017; Lee et al., 2021; Wolff and Piven, 2021), followed by decreased growth between 10 and 15 years of age (Lange et al., 2015). More specifically, it has been suggested that the social deficits seen in ASD are associated more with WM deficits in the left fronto-occipital inferior fasciculus rather than grey matter deficits (Katz et al., 2016).

Research involving neuroimaging examined the brains of infants at high familial risk for ASD and concluded that increased enlargement of the cortical surface area between 6 and 12 months of age is later followed by an overgrowth in brain size, which is perceived when these individuals are between 12 and 24 months of age (Hazlett et al., 2017). The enlargement in brain volume was observed around the same time that social defects in the children with ASD began to emerge, suggesting that an initial hyper-expansion of cortical surface areas may play a role in the development of ASD (Girault and Piven, 2020).

In adult individuals with ASD, significantly reduced WM volume was noted, allocated to 4 areas: (1) Corticospinal and cerebellar tracts, (2) frontal connections, including the uncinate fasciculus and the fronto-occipital fasciculus, (3) the internal capsule comprising nding frontostriatal and thalamocortical ascending projections, and… (4) the arcuate fasciculus connecting the Broca and Wernicke areas.

The degree of changes in brain structure was found to be correlated with the severity of the symptoms presented by the adults with ASD (Ecker et al., 2012). Nickl et al. (2012) performed a meta‐analysis of reported brain-structure changes in ASD and further highlighted age-dependent WM changes in ASD (Nickl-Jockschat et al., 2012). Specifically, their analysis confirmed reduced WM volume in several brain regions and in a cluster next to the medial ACC in young adults with ASD compared to TD (Nickl-Jockschat et al., 2012).

Advanced MRI studies using diffusion tensor imaging have allowed further insight into WM tracts in the brain (**Figure 2**). An important measurement regarding WM and myelin is fractional anisotropy (FA), which reflects the directionality of molecular displacement by diffusion. This in turn reflects fiber density and myelination in WM (Pierpaoli and Basser, 1996; Assaf and Pasternak, 2008). Many studies have taken a connectome approach, modeling pathways connecting different brain regions. This approach allows to further demonstrate structural properties of various brain structures and their impact on pathology and health (Tymofiyeva et al., 2013; Shi and Toga, 2017; Shamir and Assaf, 2025).

In relation to ASD, a study revealed that young toddlers (< 2.5 years of age) with ASD present corpus callosum (CC) tract aberrations, specifically within the pathway connecting the occipital and temporal lobes (Fingher et al., 2017). These findings showed significantly larger FA values and cross-sectional area of the temporal CC tract in the toddlers with ASD, suggesting that alterations in interhemispheric connectivity, specifically across the temporal lobes, are a characteristic of ASDs (Fingher et al., 2017). A study examined structural properties of selected neural circuits in infants at high and low familial risk for ASD, and found structural abnormalities in cerebellar and CC WM in infancy, which presented high FA values (Wolff et al., 2017). Specifically, the deficits in these WM structural properties were associated with restricted and repetitive behaviors but not social deficits. Another work that addressed the importance of WM changes and dynamics over time examined children with ASD and compared them to TD children, from 0 to 8 years of age. The study identified age-related alterations in WM tract microstructure, highlighting the evolving nature of WM in individuals with ASD (Yu et al., 2020). In contrast, a study from 2020 suggested that those tracts that are most hindered in ASD and present low FA values may not be responsible for the behavioral deficits presented in ASD (Haigh et al., 2020). Lower FA values in the anterior thalamic radiation, connecting the anterior thalamic nuclei to the PFC, have been further associated with the severity of adverse childhood experiences in individuals with ASD (Yoshikawa et al., 2022), suggesting that emotional abuse and neglect during childhood could be clinically important for WM development in ASD.

Taken together, the body of evidence on WM alterations in ASD underscores a high degree of heterogeneity across individuals, brain regions, developmental stages, and measurement modalities. While several studies report consistent reductions in WM volume or myelination in key social and language-related tracts, others show region-specific increases or age-dependent fluctuations in structural properties such as FA. These divergent findings may be attributed to methodological variability, including sample sizes, imaging techniques, and participant stratification, as well as to the inherent clinical heterogeneity of ASD, which spans a wide spectrum of symptom severity, comorbidities, and genetic backgrounds. Moreover, developmental timing appears to be a critical factor, as some WM alterations may emerge early in life while others evolve dynamically into adolescence or adulthood. Rather than indicating contradictory conclusions, this variability likely reflects distinct neurobiological trajectories within the ASD population, emphasizing the need for stratified approaches in future neuroimaging research. Understanding the biological and developmental sources of this heterogeneity will be essential to identify WM-based biomarkers and therapeutic targets relevant to specific ASD subtypes.

## Contribution of White Matter Pathology to Social Behavior Anomalies in Humans

While the previous section highlighted the link between WM differences in individuals with ASD, the following section delves further into research examining how WM abnormalities are associated with social behavior.

Research in TD humans has demonstrated that variability in WM structure correlates with differences in social cognitive abilities and behaviors. For example, individuals with higher emotional empathy exhibit greater integrity of certain WM tracts: empathic concern scores are positively associated with FA in fibers linking limbic regions with perceptual and action-related areas (Parkinson and Wheatley, 2014). Such findings suggest that well-myelinated, efficient connections between emotion-processing and other brain regions support the rapid understanding of others’ feelings. Similarly, structural connectivity among social brain regions has been tied to real-world social functioning. In a diffusion MRI study of TD young people, WM microstructure in an amygdala-centered network was highly predictive of social network size. Specifically, stronger amygdala connectivity to the orbitofrontal cortex and anterior temporal lobe explained much of the individual variation in how many friends participants had (Hampton et al., 2016). These patterns imply that even within the TD population, more robustly myelinated pathways can facilitate better social integration and skills.

Developmental evidence further underscores the link between WM maturation and social cognition. A prominent example is the emergence of Theory of Mind (ToM) in early childhood – the ability to understand beliefs and perspectives of others. Longitudinal neuroimaging has shown that the breakthrough in false-belief understanding around age 4 is associated with maturation of specific WM tracts (Grosse Wiesmann et al., 2017). Notably, these WM changes predict social-cognitive development independently of general cognitive maturation. Together, these studies in TD individuals reinforce the idea that myelin content in WM is a key biological substrate supporting human social cognition and behavior, complementing evidence from clinical populations.

The association between reduced fiber density and impaired social behavior was highlighted in a study showing that adolescents and young adults with high-functioning ASD exhibited significantly reduced FA in WM underlying the temporal lobe, and that this reduction was related to their ToM impairments; however, the relatively small sample size in this study limits the generalizability of the finding (Kana et al., 2014b).

In an editorial, Kana et al. (2014a) highlight the need to focus on functional connectivity in ASD, especially regarding the heterogeneity of the disorder, when trying to elucidate the various ways the transmission between brain areas underlie the myriad symptoms. For instance, functional connectivity analysis showed significantly weaker connectivity in the ASD group, as compared to the TD group in ToM-related brain areas (Kana et al., 2014b). A different study has shown decreased structural brain network integration, while also showing connectivity abnormalities in the right caudate and right superior temporal pole in high-functioning individuals with ASD when compared to TD individuals, brain regions associated with ToM (Tolonen et al., 2023). A study done on young patients with ASD has shown an increase in frontoparietal connectivity using virtual reality treatment, correlated with a significant improvement in their cognitive-behavioral process, such as attention, visuospatial cognition, and anxiety, linking functional connectivity and behavioral symptoms (De Luca et al., 2021). To address the challenge of heterogeneity in human ASD in relation to functional connectivity, a study analyzed functional brain networks in 105 children with ASD and 102 TD children. The analysis identified two distinct ASD subtypes based on inter-individual deviations in functional connectivity. The first subtype exhibited a pattern of hypoconnectivity, while the second displayed hyperconnectivity compared to TD children. Furthermore, inter-individual deviations in functional connectivity patterns were linked to behavioral outcomes: hypoconnectivity (subtype 1) was associated with social communication impairments, whereas hyperconnectivity (subtype 2) correlated with the severity of repetitive behaviors (Guo et al., 2022). A later study, using data from the Autism Brain Imaging Data Exchange website, has shown that children with ASD show a distinct developmental trajectory of functional connectivity, when compared to TD children. Furthermore, a negative correlation between the social responsiveness scale score and the default mode network was seen in children with ASD when compared to TD (Jiang et al., 2024).

In summary, both hyperconnectivity and hypoconnectivity are observed in ASD, each suggesting altered myelination and WM formation that disrupts normal signal transmission. Thus, while myelination emerges as a plausible substrate for social communication deficits in ASD, this relationship is not yet fully understood. A critical, cautious perspective is warranted, and further research (e.g., longitudinal and etiologically stratified studies) is needed to clarify how myelin aberrations contribute to the complex social phenotype of ASD. These anomalies may be linked to the high heterogeneity of the disorder and their diverse impact on symptoms (Shan et al., 2022). However, many human-based studies on ASD fail to adequately consider the pathophysiology of the disorder, and their inclusion of broad age ranges may dilute observed effects, complicating result interpretation (Lenroot and Yeung, 2013).

To address these issues, researchers could sequence samples or group participants based on relevant genetic and non-genetic variants, offering greater insights and reducing variability (Jeste and Geschwind, 2014). Additionally, efforts to tackle the heterogeneity of ASD have led to the creation of open-source databases containing MRI and phenotypical data, which aid in identifying neurobiological subgroups (Di Martino et al., 2017).

Although human studies offer valuable insights, animal models serve as essential complements by enabling more detailed investigation into the relationship between WM and behavior. They also enable researchers to better understand the biological processes underlying ASD and to control confounding factors more effectively (Girault and Piven, 2020).

## Myelin Plasticity in Response to Social Experience in Animal Models

Multiple studies have consistently shown that subjecting animals to social isolation, especially during critical developmental periods, disrupts OL maturation and impairs healthy myelination in the brain. For example, postweaning isolation in mice leads to a reduction in OL numbers in key brain regions associated with social behavior (Guimarães et al., 2023). Likewise, isolated dogs exhibit lower expression of myelin-related genes in the PFC WM, indicating stalled OL differentiation in this region (Hong et al., 2023).

In addition to cell number and gene expression changes, social isolation causes clear structural deficits in myelin. In one rodent model, prolonged isolation of adult mice induced notable ultrastructural changes in PFC OLs and resulted in thinner myelin sheaths in isolated mice compared to group-housed controls (Liu et al., 2012). This myelin thinning was region-specific – significant in the mPFC – and was not observed in control WM regions such as the CC or cerebellum (Liu et al., 2012).

The timing of social deprivation is crucial. Early-life (juvenile) social isolation leaves a lasting “fingerprint” on OL maturation that can persist into adulthood (Makinodan et al., 2012). Mice isolated for just 2 weeks immediately after weaning, a critical period for PFC development, develop persistent myelin deficits in the PFC that cannot be fully rescued by later social reintroduction (Broek et al., 2014). During this juvenile isolation window, OLs in the PFC fail to fully mature: they display simpler morphology and produce abnormally thin myelin wraps around axons. Electron microscopy confirmed that isolated juveniles had significantly thinner myelin coatings on PFC axons compared to socially reared mice (Liu et al., 2012). Such deviations in myelin thickness are functionally important, as suboptimal myelin (either too thin or too thick) can slow nerve conduction and disrupt neural circuit communication. Notably, the adverse myelination effects occur only if isolation overlaps with the critical developmental period – if mice are isolated after this period, they do not exhibit the same long-term myelin deficits (Chen and Hong, 2018). This indicates that there is a sensitive window during which social interaction is necessary for normal OL differentiation and myelin formation; missing that window results in enduring WM abnormalities.

Mechanistically, social isolation during critical periods interferes with key molecular signals required for OL development. Juvenile mice exposed to isolation show a marked downregulation of neuregulin-1 (NRG1) in the PFC (Van Overwalle et al., 2020). NRG1 is an essential growth factor that binds to ErbB3 receptors on OLs, promoting their maturation and the formation of myelin. The isolation-induced drop in NRG1 likely impairs this signaling pathway. In fact, the consequences of social isolation can be mimicked by directly disrupting NRG1–ErbB3 signaling. Experimental studies demonstrated that knocking out *ErbB3* in OLs produces the same outcome as social isolation. Mice lacking OL-specific ErbB3 showed hypomyelination in the mPFC and abnormal social behaviors even when raised in a normal social environment (Makinodan et al., 2012). This finding underscores that NRG1–ErbB3 signaling in oligodendroglia is a critical mediator between social experience and myelination.

Encouragingly, some studies indicate that myelin deficits and their associated behavioral changes are at least partially reversible under the right conditions. One key experiment demonstrated that the mode of re-socialization after isolation can determine the extent of recovery in both myelin and behavior. Makinodan et al. (2017) took juvenile mice that had been socially isolated during the critical period and then gave them different social housing interventions. Remarkably, previously isolated mice that were reintroduced into a normal social group with continuously socialized (group-housed) peers showed a restoration of normal myelin thickness in the mPFC, so that their myelin levels essentially “caught up” to those of control mice that had never been isolated (Makinodan et al., 2017). Alongside this structural recovery, the social behavior of these mice also normalized: their social interaction frequency became comparable to control levels (Makinodan et al., 2017). In contrast, isolated mice that were only re-housed with other formerly isolated mice did not show the same improvements – they continued to exhibit thinner myelin in the mPFC and persistent social interaction deficits despite resocialization with their similarly isolated peers (Makinodan et al., 2017). This experiment elegantly underscores that social stimuli can actively influence OL function, and the quality of social experience is critical for harnessing this form of brain plasticity.

Beyond environmental interventions, direct myelin-targeted therapies have shown parallel results, further highlighting the behavior-myelination interplay. In adult mice that experienced chronic social isolation, researchers tested whether promoting OL maturation pharmacologically could improve behavior. The drug clemastine, an antimuscarinic known to enhance OL differentiation and myelination, was administered to isolated adult mice for a brief period (Liu et al., 2016b). Strikingly, clemastine-treated isolated mice showed reversal of their social avoidance behavior – they became more sociable and showed reduced anxiety-like traits, approaching the behavior of non-isolated mice (Liu et al., 2016b). This behavioral rescue was accompanied by a rescue of myelination in the PFC: previously isolated mice displayed increased myelin thickness and OL activity after clemastine treatment (Liu et al., 2016b). Together, this provides strong evidence of a causal link: the myelin deficits in PFC are a key contributor to the social and emotional abnormalities seen in isolated animals, and repairing those deficits can substantially improve outcomes.

Interestingly, changes related to PFC myelination have been linked to epigenetic changes specifically in OLs and in genes relating to their differentiation and proliferation, such as *Mbp*, *Sox10*, and *Olig2*. These changes are induced through chromatin regulation, primarily via histone modifications; however, their direct contribution to social behavior remains to be fully demonstrated (Huynh and Casaccia, 2010; Liu et al., 2016a, 2018; Samudyata et al., 2020).

Further still, DNA methylation of OL precursor cells (OPCs) differentiation into OLs plays a key role in developmental myelination, with hypermethylation at promoter regions causing decreased expression during OPC differentiation, and vice versa in the beforementioned genes (Moyon and Casaccia, 2017).

## Myelin-related Abnormalities in Autism Spectrum Disorder Rodent Models

Animal studies have provided valuable insight into genetic-based ASD and the associated social behaviors (Moy et al., 2004; Bohlen et al., 2023; Crawley, 2023; Fischer et al., 2024), and some also further delved into the relationship between social behavior and myelin properties.

Individuals afflicted with Pitt-Hopkins syndrome, an ASD resulting from autosomal dominant mutations in the human transcription factor 4 gene (*TCF4*), are known for their autistic-like behavior and avoidance of social interaction (Li et al., 2019). Pitt-Hopkins syndrome has been further associated with decreased numbers of OLs and impaired myelination when compared to TD controls (Phan et al., 2020; Chen et al., 2021). Recent research found that clemastine, known to promote myelination by antagonizing muscarinic M1 receptors, thereby enhancing the differentiation of OPCs into mature, myelinating OLs (Mei et al., 2014), was able to restore myelination properties and behavior, although social behavior was not assessed in this mouse model of Pitt-Hopkins syndrome (Bohlen et al., 2023). These findings offer pre-clinical evidence suggesting a possible treatment to improve postnatal myelination using pharmacological means in NDDs (Barak et al., 2019; Bohlen et al., 2023; Rokach et al., 2024).

*SHANK3*, a gene encoding a postsynaptic scaffolding protein, is strongly implicated in ASD and is considered a high-risk gene (Monteiro and Feng, 2017). Mutations in *SHANK3* are present in approximately 1% of ASD cases (Costales and Kolevzon, 2015). Studies on *Shank3* mutations have reported impaired behavior, along with structural, WM, and myelin abnormalities (Delling and Boeckers, 2021; Wang et al., 2021; Fischer et al., 2024). These findings have been further validated in human studies, which revealed WM alterations in participants with *SHANK3* mutations, particularly affecting long association fiber tracts such as the uncinate fasciculus and the inferior fronto‐occipital fasciculus (Jesse et al., 2020). However, due to the small number of participants and variability in the genetic conditions associated with *SHANK3*, these results may not be generalizable.

A recent study utilizing *Shank3*-mutant mouse model established a link between the mutation and myelin deficits; however, it did not investigate behavioral outcomes or the relationship between myelination deficits and social behavior. Mutant mice exhibit a reduced CC volume, consistent with observations in individuals with Phelan-McDermid syndrome, as well as decreased spinal cord myelination compared to non-mutant control mice (Malara et al., 2022). Furthermore, in human induced pluripotent stem cell (iPSC)-derived cerebral organoids, Malara et al. (2022) demonstrated a reduced number of myelin basic protein–positive cells in SHANK3-mutant lines, suggesting a causative role for the mutation in myelin deficits.

Our lab recently reported further findings demonstrating how mutated *Shank3* affects myelination and the consequent WM formation (Fischer et al., 2024). This study was focused on a *Shank3* deficient mice carrying an insertion mutation (*InsG3680*), which is homologous to the one found in humans (Zhou et al., 2016; Ivashko-Pachima et al., 2022). Mutant mice exhibit reduced connectivity between brain regions, compromised myelin ultrastructure, and motor skill deficits compared to control littermates. They also show reduced expression of key genes associated with OL differentiation and morphology. Interestingly, while the number of OLs increased, the expression of myelination-related genes is significantly reduced (Fischer et al., 2024). Primary OPC cultures derived from these mice demonstrate lower calcium activity in response to glutamate, a finding similarly observed in iPSCs carrying the same mutation. Notably, restoring *SHANK3* expression in mouse-derived OPCs using a plasmid containing the full-length human *SHANK3* isoform led to increased expression of postsynaptic proteins, suggesting a potential pathway for therapeutic intervention (Fischer et al., 2024). However, whether restoring myelination properties in this mouse model improves behavior in general, and social behavior specifically, remains to be further investigated.

Haploinsufficiency of the autism-associated gene *ANKS1B* leads to a syndromic NDD that includes social behavior deficits (Carbonell et al., 2019). *Anks1b*-deficient mice display marked deficits in OL maturation and central nervous system myelination, including reduced OL abundance and myelin in the CC (Cho et al., 2023). These WM abnormalities, observed also in patients with *ANKS1B* haploinsufficiency, are accompanied by autism-like behavioral impairments, notably reduced social interaction in the mutant mice (Cho et al., 2023). Consistently, restricting the *Anks1b* mutation to OL-lineage cells recapitulates the same social and sensory deficits, whereas deleting *Anks1b* in neurons does not produce these behavioral changes, demonstrating that the phenotypes stem specifically from OL dysfunction (Cho et al., 2023). Notably, treatment with clemastine reverses the social deficits in adult *Anks1b*-mutant mice, showing that enhancing OL function can rescue these behavioral abnormalities (Cho et al., 2023).

Additional study demonstrating ASD heterogeneity examined 26 mouse models of ASD using MRI (Ellegood et al., 2015). The research has shown that while there is not a single neuroanatomical pattern defining ASD in mice across the different models, there are major overlaps between them. Models of *Shank3*, *Fmr1*, *En2*, and *Nrxn1a* mutations were noted to have an increase in several WM structures, most notably the CC, fimbria, fornix, and parieto-temporal lobe, while a decrease was found in the cerebellar cortex. A different cluster, consisting of *And*R, BTBR, *Gtf2i* (dp/dp), *Itgβ3*, 15q11–13, *Slc6A4* KI (129), and *Nlgn3* KI models, showed a decrease in the cerebral peduncle, CC, internal capsule, globus pallidus, hippocampus, and striatum. The last group, consisting of 16p11, BALB/c, *Cntnap2* (−/−), *Gtf2i* (+/−), *Mecp2*, *Slc6A4* KI (B6), *Slc6A4* KO, and XO, showed a decrease in frontal and parietotemporal lobes size and an increase in cerebellum size. Although these groups are not behaviorally clustered and the contribution of these WM abnormalities to behavior was not tested, the findings further highlight the neuroanatomical diversity of ASDs (Ellegood et al., 2015).

Work done on BTBR mouse model for ASD, known for its impaired social behavior (Scattoni et al., 2013; Meyza and Blanchard, 2017) showed reduced FA at the CC as well as increased volume in several regions correlating with reduced social behavior, including the anterior commissure, hypothalamus, olfactory bulbs, and the pons (Ellegood et al., 2013). A different study has replicated the neuroanatomical findings in the CC, as well as found an abnormal inter-hemispheric commissure in the rostral region of the third ventricle (Kerever et al., 2015).

Another rodent model used in ASD research is one exposed to valproic acid (VPA), a compound known to increase the risk of ASD in children. VPA exerts its effects primarily through inhibition of histone deacetylases, leading to increased histone acetylation and altered gene expression, including in pathways regulating neurodevelopment and OL differentiation. These animals exhibit behavioral phenotypes that closely resemble those observed in humans with ASD (Nicolini and Fahnestock, 2018). This model provides valuable insights into environmentally - or epigenetically - driven ASD, as opposed to the genetically engineered models mentioned above. Research has shown that the model exhibits WM-related changes, including reduced myelin content in brain regions associated with social behavior, such as the mPFC, basolateral amygdala, and piriform cortex. However, a causal relationship between these WM deficits and social behavior impairments was not investigated. Additionally, the model demonstrated reduced myelin thickness, and fewer OLs and OPCs compared to saline-treated controls. However, no significant effects on histone modifications were observed in any of the studied regions (Graciarena et al., 2019). A study on juvenile rats exposed to VPA showed myelination deficits in the CC, including reduced myelin basic protein expression, a lower proportion of myelinated axons, and abnormal, non-compact myelin ultrastructure. These changes were accompanied by a decrease in OLs and an increase in OPCs. Notably, such alterations were not observed in the mPFC or somatosensory cortex. In younger, infant rats, the CC also displayed hypomyelination; however, the numbers of OLs and OPCs remained unchanged (Uccelli et al., 2021).

Interestingly, Pazhoohan et al. (2014) reported a pro-myelinating effect of VPA under certain conditions. For example, in demyelinating disease models, VPA treatment can enhance remyelination by recruiting neural stem cells and OPCs to lesions. These seemingly conflicting findings likely reflect context-dependent effects of VPA: early developmental exposure may disrupt normal myelination (as seen in ASD models), whereas postnatal or adult administration (often in injury or disease contexts) can engage VPA’s epigenetic actions to facilitate OL differentiation and myelin repair (Pazhoohan et al., 2014).

## Discussion

The broad term of ASD encompasses many different afflictions and implications, undermarking it as a complex and heterogeneous condition. As a result, the current understanding of the interplay between WM properties and social behavior in ASD remains complex and marked by discrepancies, which can be attributed to several non-mutually exclusive factors. Methodological differences are one key consideration: studies vary in neuroimaging modality (structural MRI *versus* diffusion tensor imaging *versus* advanced diffusion models), analytical approach, sample size, and statistical power, all of which can influence whether WM anomalies are detected (Thompson et al., 2020). Biological heterogeneity within ASD is another likely contributor – autism is a spectrum with diverse genetic and neurobiological underpinnings, so WM pathology may manifest only in subgroups of individuals (DiPiero et al., 2023). Comorbid conditions such as epilepsy and anxiety further complicate the picture. Epilepsy in particular is known to affect myelinated networks; for example, rodent ASD models with seizure activity show secondary demyelination (Galvez-Contreras et al., 2020), potentially confounding human neuroimaging results if such factors are not controlled. Finally, the timing of assessments is critical. WM differences in ASD appear to be dynamic across development (Thompson et al., 2020) – an anomaly detectable in childhood (when myelination is rapidly changing) might diminish or reverse by adulthood, or vice versa. A nuanced interpretation that accounts for these factors is therefore essential.

ASD etiology varies greatly and can be related to genetics (Huguet et al., 2013), environment (Modabbernia et al., 2017), immunological considerations (Estes and McAllister, 2016), sex (Ferri et al., 2018), and more. This heterogeneity is evident in the wide variability seen in terms of cognitive function, language skills, social communication abilities, and sensory processing profiles across individuals with ASD (Masi et al., 2017). However, this distinction is often overlooked in research. Few studies specifically focus on a particular type of ASD, and many fail to report findings in this context. Understanding the factors contributing to the underlying causes and heterogeneity of ASD and identifying meaningful sub-types within ASD are ongoing challenges (Feczko and Fair, 2020). Meeting these challenges will require researchers to navigate the vast diversity associated with ASD and account for the idiosyncrasies inherent in each case.

The existence of comorbid conditions further adds to the complexity of studying the neuroscience of ASD. As many individuals with ASD have comorbid medical or psychiatric conditions, such as epilepsy, anxiety, or gastrointestinal issues (Al-Beltagi, 2021), understanding the relationships between ASD and these comorbidities, as well as their impacts on overall well-being and quality of life, is needed. Finally, the diagnostic criteria for ASD have evolved over time, leading to differences in case selection and diagnostic criteria across studies (Smith et al., 2015).

It should also be noted that whereas differences in brain connectivity, structure, and function have been observed in individuals with ASD, the specific neurobiological mechanisms underlying these differences and their relationship to ASD symptoms are not yet fully understood. The use of advanced imaging techniques and molecular studies is necessary to uncover the neural and glial underpinnings of ASD. Moreover, such experiments should address specific sub-groups of ASDs, as done in animal-model studies, rather than consider the wide arc of the disorder. Such challenges necessitate the adoption of more robust research designs, including large sample sizes, stratification based on sub-groups, longitudinal research design, and the integration of multiple assessment measures connecting structure to behavior as suggested in Müller and Fishman’s review (Müller and Fishman, 2018).

Furthermore, recent advances in machine learning, big data analytics, and omics technologies offer promising tools to address the challenges posed by heterogeneity (Szoko et al., 2017; Lombardo et al., 2019).

Overall, myelin and WM abnormalities appear to be prevalent in ASDs, with mechanisms spanning genetic, molecular, and cellular levels. Further exploration of these differences and a deeper understanding of the underlying changes at the genetic, molecular, and cellular levels could enhance our knowledge of the pathways involved in both health and disease, ultimately improving prospects for developing effective treatment options. As myelination is a developmental process, occurring in early postnatal stages, early diagnosis of ASD can crucially affect the treatment administered (Magiati et al., 2014). A better understanding of the myriad genomic and epigenetic landscapes of ASD could lead to quicker, more robust interventions, including the usage of micro and nanoparticle systems (Rokach et al., 2024).

It should also be acknowledged that the sometimes-conflicting direction of findings in human-based research is related more to the heterogeneity of the diverse syndromes. Further developing better, more accurate human-like models could lead to better clinical avenues, with iPSCs and organoids becoming more prevalent and easier to maintain (Turhan et al., 2021).

In summary, a profound link can be drawn between WM abnormalities and ASDs, stemming from differing or convergent genetic and idiopathic mechanisms. Acknowledging this effect could lead to better therapeutic targets and improved care for individuals with ASD, while work must be done to further untangle the differences in ASD pathology.

## Data Availability

*Not applicable.*
